# Applying Entropic Measures, Spectral Analysis, and EMD to Quantify Ion Channel Recordings: New Insights into Quercetin and Calcium Activation of BK Channels

**DOI:** 10.3390/e27101047

**Published:** 2025-10-09

**Authors:** Przemysław Borys, Paulina Trybek, Beata Dworakowska, Anna Sekrecka-Belniak, Michał Wojcik, Agata Wawrzkiewicz-Jałowiecka

**Affiliations:** 1 Department of Physical Chemistry and Technology of Polymers, Silesian University of Technology, Strzody 9, 44-100 Gliwice, Poland; przemyslaw.borys@polsl.pl; 2 Institute of Physics, University of Silesia in Katowice, 75. Pułku Piechoty 1, 41-500 Chorzów, Poland; paulina.trybek@us.edu.pl; 3 Institute of Biology, Department of Physics and Biophysics, Warsaw University of Life Sciences, Nowoursynowska 159, 02-787 Warsaw, Poland; beata_dworakowska@sggw.edu.pl (B.D.); anna_sekrecka-belniak@sggw.edu.pl (A.S.-B.); 4 Faculty of Biomedical Engineering, Silesian University of Technology, Roosevelta 40, 41-800 Zabrze, Poland; mw302051@student.polsl.pl

**Keywords:** patch-clamp, BK channel, channel gating, stimulus-specific gating patterns, sensors coupling, empirical mode decomposition, spectral analysis, information entropy

## Abstract

Understanding the functional modulation of ion channels by multiple activating substances is critical to grasping stimulus-specific gating mechanisms and possible synergistic or competitive interactions. This study investigates the activation of large-conductance, voltage- and Ca^2+^-activated potassium channels (BK) in the plasma membrane of human bronchial epithelial cells by Ca^2+^ and quercetin (Que), both individually and in combination. Patch-clamp recordings were analyzed using open state probability, dwell-time distributions, Shannon entropy, sample entropy, power spectral density (PSD), and empirical mode decomposition (EMD). Our results reveal concentration-dependent alterations in gating kinetics, particularly at a low concentration of quercetin ([Que] = 10 μM) compared with [Que] = 100 μM, where some Que-related effects are strongly attenuated in the presence of Ca^2+^. We also identify specific frequency bands where oscillatory components are most sensitive to the considered stimuli. Our findings highlight the complex reciprocal interplay between Ca^2+^ and Que in modulating BK channel function, and demonstrate the interpretative power of entropic and signal-decomposition approaches in characterizing stimulus-specific gating dynamics.

## 1. Introduction

Ion channels are membrane proteins that enable the rapid and selective passage of ions, such as Na^+^, K^+^, Ca^2+^, or Cl^−^, down their electrochemical gradients [1]. Due to their role in regulating cellular ion homeostasis and signaling, they are important molecular drug targets [2,3,4,5,6,7,8]. From the perspective of modern channel-oriented pharmacology, a proper understanding of the conformational dynamics of ion channels is paramount.

The primary experimental method used to study the functional characteristics of ion channels is the patch clamp technique [9,10]. This electrophysiological technique enables the direct measurement of ion flow through individual channels under controlled conditions in real time. Often, the results take the form of a time series of ionic currents through one or more ion channels. When recorded in single-channel mode, these time series capture the conformational dynamics of individual channel proteins. Based on the signal amplitudes, one can recognize the conducting (open) and non-conducting (closed) states of the channel [11,12]. Furthermore, one may describe the signal using some informative metrics, including the probability of the channel being in its open state ($p_{op}$), single-channel conductance, and the parameters of the opening and closing kinetics (e.g., average dwell times and the rate constants of open-closed and closed-open transitions) [9]. These parameters provide elementary insights into the dynamics of open/closed channel fluctuations under given conditions (gating). However, a detailed picture of the channel gating process remains elusive. For example, several key aspects remain unresolved: the exact number of stable channel conformations and their interconnectivity [11,13,14,15,16], the detailed principal molecular mechanisms responsible for ionic permeation [17,18,19], and the structure–function relationships [20,21,22,23]. Among the poorly understood aspects of channel conformational dynamics are the existence of stimulus-specific gating patterns, their inherent timescales, and their possible mechanistic origins.

Most reports in the literature indicate the existence of a finite number of stable open/closed states that can represent the conformational dynamics of many ion channel types [19,24,25,26,27,28,29,30,31]. However, it remains largely unknown how the binding of specific channel-modulating molecule(s) affects the channel’s conformational dynamics, or whether it induces particular changes in the stability, number, or interconnectivity of the channel’s predefined stable states (conformations). Additionally, it is unclear how different channel-modulating substances might compete or exert synergistic effects on channel-gating dynamics when present simultaneously in the channel’s microenvironment. Addressing these questions is challenging due to existing computational constraints, as it requires long-term simulations that often exceed the capabilities of prevailing techniques such as Molecular Dynamics, even in the approximate variants like the extrapolated motion technique, designed for long-term simulations [32,33]. This situation necessitates the adoption of alternative methods for analyzing stimulus-specific changes in channel gating. In this context, nonlinear signal analysis methods applied to patch-clamp data emerge as a possible solution.

As mentioned above, patch-clamp data provide real-time recordings that depend on conformational changes in ion channels. A stimulus, such as a channel-activating biochemical agent, that alters the channel’s structure and energetic landscape, will also significantly affect features of the corresponding patch-clamp signals. Furthermore, the corresponding changes in conformational dynamics can be stimulus-specific to some extent and can therefore serve as determinants of specific channel–agent or inter-agent interactions. Patch-clamp data have a complex and noisy nature stemming from the nonlinear processes that govern channel gating. Thus, the reliable recognition of the hypothetic stimulus-specific signal features and the symptoms of inter-agent rivalry/cooperation in channel activation is practically unattainable by visual inspection or simple analytic approaches, including $p_{op}$ evaluation (which, being a cumulative measure, may take similar values despite the changes in conformational switching). In this context, entropy-based measures, spectral analysis, and signal decomposition algorithms provide suitable methodologies for capturing and quantifying signal complexity across multiple time scales, which can then be interpreted in terms of the kinetic and, potentially, physical components of channel gating.

In this work, we consider the utility of the information entropy metrics (Shannon entropy and sample entropy), spectral analysis, and signal decomposition using Empirical Mode Decomposition in capturing the stimulus-specific features of channel gating and symptoms of inter-agent interactions between two different channel-activating substances. As a running example, we take the activity of the large-conductance voltage- and Ca^2+^-activated K^+^ channel (BK) [24,34,35] at various concentrations of Ca^2+^ and quercetin (Que). Each substance acts as an effective BK channel activator [24,34,36,37,38,39,40,41,42]. However, the potential cumulative effects of these substances, as well as the distinct Que-specific and Ca^2+^-specific features of the corresponding single-channel patch-clamp signals, have not yet been investigated.

BK channels are potassium channels characterized by a large single-channel conductance (150–300 pS) [43]. They regulate physiological processes that require rapid changes in membrane potential, such as neural transmission, hearing, endocrine secretion, and smooth muscle contraction [36,44,45,46,47,48]. Structurally, BK channels are tetramers. Each monomer is composed of transmembrane helices that form a pore-gate (PG) domain and a voltage-sensing domain (VSD). Each monomer also has a cytoplasmic C-terminal domain (gating ring, GR), which is mainly responsible for Ca^2+^-sensing. The three types of functional domains—voltage sensors, pore-gate domains, and Ca^2+^ sensors—operate independently but are coupled allosterically. This means that changes in one domain may evoke conformational rearrangements in the others. Quercetin (2-(3,4-dihydroxyphenyl)-3,5,7-trihydroxychromen-4-one) is a flavonoid known for its multi-target action, which results in global anti-oxidative, anti-inflammatory, anti-mutagenic, and anti-carcinogenic activity [49]. It promotes the open state of the BK channel after its binding to the channel pore [41]. However, quercetin binding to the BK channel’s gating ring cannot be excluded. Additionally, quercetin can activate the BK channel indirectly by interacting with the cell membrane [50], where it affects lipid packaging, fluidity, and permeability [51,52,53].

In this work, we extend the existing literature by providing a basic kinetic description of the individual and combined effects of Que and Ca^2+^ on BK channel activity and by conducting a comprehensive analysis of the properties of the corresponding signals using selected spectral and entropic methods. These methods should provide information about the specific frequency bands, where oscillatory signal components are most sensitive to the combined effects of the analyzed stimuli. We not only present the raw results and highlight the differences, but also aim to initiate the development of an interpretative framework for applying these computational methods to the analysis of single-channel recordings.

## 2. Materials and Methods

### 2.1. Cell Culture and Electrophysiology

Human bronchial epithelial cells were cultured under standard conditions in minimum essential Eagle’s medium (Sigma Aldrich, Darmstadt, Germany) supplemented with 10% fetal calf serum, 100 units/mL penicillin, and 100 lg/mL streptomycin (PAA) at 37 °C in a humidified atmosphere containing 5% CO_2_. The cells were fed and passaged every 3–4 days.

For the patch-clamp recordings, the culture medium was replaced with a control solution comprising 130 mM potassium gluconate, 2 MgCl_2_, and 0.05 EGTA (pH 7.3–7.4). BK channel activation was induced using Ca^2+^ and quercetin via a perfusion system. CaCl_2_ and/or quercetin (Sigma Aldrich, Darmstadt, Germany) were added to the control solution to reach the desired concentrations. Recordings were performed at room temperature (22 °C) using a borosilicate glass pipette (Harvard Apparatus, Holliston, MA, USA) with a resistance of 7–20 MΩ. Experiments were carried out in the inside-out single-channel patch-clamp configuration using Axopatch 200B amplifier (Molecular Devices, LLC., San Jose, CA, USA). The signals were low-pass filtered at 1 kHz and digitized at 10 kHz (i.e., sampled every 100.00 μs) with the Clampex 10 acquisition software (Molecular Devices, LLC., San Jose, CA, USA). The pipette potential was held at +50 mV throughout all recordings. The measurement error for single-channel currents was determined by the amplifier specifications ΔI = 1 $\times \;10^{-6}$ pA. For each tested Ca^2+^ and Que concentration, single-channel current time series were recorded from three to fifteen independent patches, a sample size typical of ion channel electrophysiology studies. Each experimental time series comprised at least $N_{I}$ = $2\;\times \;10^{5}$ current values with maximum lengths reaching up to $N_{I}$ = $1.1\;\times \;10^{6}$. Most recordings exceeded $N_{Icell.}$ = $8\;\times \;10^{5}$ values, corresponding to typical dwell-time series lengths of approximately ∼$2\;\times \;10^{5}$. As can be seen, the obtained patch clamp recordings vary in length. Consequently, individual experimental series provide different amounts of information about the underlying gating dynamics, which can bias the results of the nonlinear analysis. From this perspective, when needed (e.g., for SampEn evaluation or EMD), the original recordings were divided into subseries (windows) of *n* = 100,000 length (in the case of single-channel currents) and $n_{dw}$ = 4000 length (in the case of dwell-time series).

### 2.2. Open State Probability and Dwell-Time Characteristics

The open-state probability ($p_{op}$) is the most commonly evaluated parameter used to describe single-channel patch-clamp data. It is defined as the fraction of data points assigned to the open state relative to the total number of points sampled in the time series. Its calculation is straightforward; however, the channel’s functional states (open vs. closed) must be identified first. For this purpose, a threshold current must be established to distinguish open from closed states. In this study, the threshold current was determined using the standard half-amplitude method [9,11]. After identifying the open and closed states, dwell-time series were generated by measuring the durations of successive channel states. Accordingly, two types of input data were analyzed in this study: raw recordings (time series of single-channel currents) and reduced data (dwell-time series).

## 3. Theoretical Methods

### 3.1. Entropic Analysis

Shannon’s pioneering concept of characterizing a signal through its information content laid the foundation for a wide range of subsequent advances in complexity analysis [54]. Over time, this framework was substantially expanded to include diverse measures of complexity, including spectral entropy [55], Rényi entropy [56], approximate entropy [57], sample entropy [58], or their multiscale version in the form of multiscale entropy [59]. Each of these extension approaches offers unique insights into the underlying structure and dynamics of signals, capturing different aspects of randomness, predictability, and organization. In this study, we investigate two distinct classical, information, entropy-based characterizations of the signal: Shannon entropy, which describes the complexity of the distribution of a given variable, and sample entropy, which quantifies the dynamical complexity of sequences of that variable and the tendency for patterns to occur within these sequences [60,61,62].

Shannon entropy of a signal *X* quantifies the uncertainty associated with its distribution over *k* possible states. Given a probability distribution p(X), where $p_{k}$ represents the probability of the system being in the *k*-th state, the Shannon entropy $H_{X}$ is defined as

(1) $$H_{X}=-\sum_{k}p_{k}\log\left(p_{k}\right),$$

which corresponds to the expected value of the negative logarithm of the state probabilities. In information theory, using the logarithm of base 2 provides the average number of bits to encode symbols of signal *X*. The possibility of quantifying this measure in bits is of enormous importance, as it allows for the standardization of similar measurements between various ion channels. In this work, Shannon entropy is calculated to characterize the complexity of the dwell-time distributions of BK channel states, as well as for the two-dimensional distributions of the frequency and squared amplitude of the clustered empirical mode decomposition results (described later in the Section 3.3).

The interpretation of this measure depends upon the application. In the case of measuring Shannon entropy for the dwell-time series, it quantifies the probability distribution of the dwell times: the less regular the dwell times in channel switching, the larger the Shannon entropy. Thus, this can be linked to the number of conformational states which are active in switching, with each of them contributing a different time scale to the dwell time distribution. A straightforward inference is that a stable open or a stable closed conformation should yield a very low Shannon entropy, as it is dominated by long dwell times for the favored conduction state, and only short dwell times are associated with the opposite state. In such cases, intermediate dwell times occur with near-zero probability. On the other hand, Shannon entropy increases in partially activated states, where multiple open and closed conformations contribute to the overall recording, and the distributions of both open and closed intervals, are significantly broadened. Such an entropic approach allows us to quantify the complexity of the switching, which is otherwise difficult to see with the naked eye (e.g., regular switching patterns involving few conformations may look similar to irregular patterns involving many conformations, yet they differ significantly in their entropy value).

The application of entropy to the spectral parameters takes advantage of this measure to quantify the variability of a time series in well-defined units. We slide an observation window (*m* samples in length for a series of length N>m) over the investigated series, calculate the spectrum, and investigate the variability of the low-, intermediate-, or high-frequency content. Again, we can draw conclusions for the stable open or closed states, where we expect the small frequency peaks to attain high and repeating values.

Sample entropy (SampEn) [58] quantifies the regularity and complexity of a time series. Given a discrete time series $X={\left\{x_{n}\right\}}_{n=1}^{N}$ consisting of *N* data points, we construct a sequence of *m*-dimensional (m<N) embedding vectors $u_{m}\left(i\right)=\left\{x_{i},x_{i+1},\ldots ,x_{i+m-1}\right\}$ for i=1,…,N−m+1.

The dissimilarity between two vectors $u_{m}\left(i\right)$ and $u_{m}\left(j\right)$ is measured using the Chebyshev distance:

(2) $$d\left[u_{m}\left(i\right),u_{m}\left(j\right)\right]=\max_{k=0,\ldots ,m-1}\left(|x_{i+k}-x_{j+k}|\right).$$

Since the probability of an exact match between vectors in empirical data is negligible, a similarity threshold *r* must be introduced. Typically, this is chosen heuristically as 10% to 20% of the standard deviation σ of the signal. Two vectors are considered similar if $d[u_{m}\left(i\right),u_{m}\left(j\right)]\leq r$; otherwise, they are treated as dissimilar.

The probability $C_{i}^{m}\left(r\right)$ that a given template vector $u_{m}\left(i\right)$ is similar to any other vector is defined as

(3) $$C_{i}^{m}\left(r\right)=\frac{n_{i}^{m}\left(r\right)}{N-m},$$

where $n_{i}^{m}\left(r\right)$ is the number of vectors $u_{m}\left(j\right)$ satisfying $d[u_{m}\left(i\right),u_{m}\left(j\right)]\leq r$ for j≠i.

The mean probability of similarity across all template vectors is given by

(4) $$C^{m}\left(r\right)=\frac{1}{N-m+1}\sum_{i=1}^{N-m+1}C_{i}^{m}\left(r\right).$$

Finally, the sample entropy (SampEn) is defined as the negative natural logarithm of the conditional probability that two sequences that are similar for *m* points remain similar for m+1 points:

(5) $$\mathrm{SampEn}\left(m,r,N\right)=-\log\left(\frac{C^{m+1}\left(r\right)}{C^{m}\left(r\right)}\right).$$

Here, we can provide an interpretation of this measure. $C^{m}\left(r\right)$ is related to the probability of finding repeating sequences of length *m* in the recording. Among the sequences of length m+1, which contribute to $C^{m}\left(r\right)$, some will repeat on position m+1 and some will not. We could write symbolically that $C^{m+1}\left(r\right)/C^{m}\left(r\right)∼p\left(m+1\right)/p\left(m\right)=p\left(m+1|m\right)$, and consider this as a kind of conditional probability for a pattern of length *m* that continues to length m+1.

Throughout this study, SampEn is calculated for the dwell-time series of channel states with the embedding dimension m=2 and the similarity threshold r=0.2σ. The same similarity threshold was also used in previous studies, where the data describing ion channel activity were analyzed [60,61]. In this approach, we can consider SampEn as a measure of average connectivity of a conformational state (which exhibits a particular characteristic dwell time) to other conformational states (exhibiting their own characteristic dwell times). The broader the dwell time distribution of the target states, the more bits are required to distinguish them, and the larger the SampEn. In a Markov chain graph [24], we could link this to the average number of connections between nodes of the graph.

In Appendix A, we provide an extended explanation to facilitate a deeper understanding of the Shannon entropy and sample entropy analyses of the dwell-time series.

### 3.2. Spectral Analysis

The power spectrum density (P([Que],[Ca],f)) was calculated using the Fast Fourier Transform (FFT) for each patch-clamp current series. Then, to describe the distribution of the signal power across different frequencies under particular Que and Ca^2+^ stimulation, the median power spectrum was calculated from the power spectra obtained from all available recordings at a fixed Que and Ca^2+^ concentration.

Because analyzing the simple power spectrum plot can be difficult due to the large range of values in the overall signal, the results can be replotted with respect to the power spectrum of the unstimulated channel:

(6) $$\hat{P}\left(\left[Que\right],\left[Ca\right],f\right)=\frac{P([Que],[Ca],f)}{P(0,0,f)}$$

In other words, through the introduction of $\hat{P}\left(\left[Que\right],\left[Ca\right],f\right)$, one can observe the ratio by which the peak grows or decreases (twofold, threefold, etc.) in response to the activating agent, here Que or Ca^2+^.

Because the frequency domain can capture rich information about the underlying dynamics, we also aimed to characterize the temporal variability of power spectral peaks associated with specific activation types.

For this purpose, we applied a windowed Fourier Transform to temporal fragments of the signal (Hamming window, width 12.8 ms). This window length was chosen as a compromise: it preserved most spectral features considered important for the full recording while providing sufficient resolution to focus on individual conformations of the Markov chain nodes. For example, a low-frequency peak may correspond to a closed state (low peak value) or to an open state (high peak value). Thus, there will be some distribution of peak values associated with the diversity of channel states available under a particular stimulation.

The algorithm used to obtain the power spectra at successive time points in terms of the BK channel activation by Que and/or Ca^2+^ can be formulated as follows:

1. Concatenate all recordings at a given [Que] and [Ca] into the vector $\vec{x}\left(\left[Que\right],\left[Ca\right]\right)$.
2. Set n=0.
3. Copy the sub-vector $\left\{x_{n},\ldots ,x_{n+W}\right\}$ into a *W*-element vector ${\vec{y}}_{n}$, multiplying by the Hamming window (to avoid frequency artifacts due to windowing at window edges)
  (7) $$\begin{array}{c} y_{i}\left(\left[Que\right],\left[Ca\right],n\right)=x_{n+i}\left(\left[Que\right],\left[Ca\right]\right)\left[a_{0}-\left(1-a_{0}\right)cos\frac{2\pi i}{W}\right], \end{array}$$
  (8) $$\begin{array}{c} i=0,\ldots ,W-1,\;a_{0}=\frac{25}{46} \end{array}$$
4. Find the power spectrum of $\vec{y}\left(\left[Que\right],\left[Ca\right],n\right)$, $\vec{P}\left(\left[Que\right],\left[Ca\right],n\right)$.
5. If $n<len(\vec{x}\left(\left[Que\right],\left[Ca\right]\right)-W-1$, n=n+1, go to step 3.

Having all the power spectra vectors $\vec{P}$, calculate the mean and standard deviation of each peak with respect to n (in order to establish histogram intervals to characterize all samples):

(9) $$\begin{array}{c} \bar{P}=\frac{1}{\sum_{\left[Que],[Ca\right]}len(\vec{x}\left(\left[Que\right]\left[Ca\right]\right)}\sum_{\left[Que][Ca\right]}\sum_{n=0}^{len(\vec{x}\left(\left[Que\right],\left[Ca\right]\right)}P_{i}\left(\left[Que\right],\left[Ca\right],n\right) \end{array}$$

(10) $$\begin{array}{c} \sigma_{P}^{2}=\frac{1}{\sum_{\left[Que],[Ca\right]}len(\vec{x}\left(\left[Que\right]\left[Ca\right]\right)}\sum_{\left[Que][Ca\right]}\sum_{n=0}^{len(\vec{x}\left(\left[Que\right],\left[Ca\right]\right)}{\left(P_{i}\left(\left[Que\right],\left[Ca\right],n\right)-{\bar{P}}_{i}\right)}^{2} \end{array}$$

For each peak in frequency *i*, create a histogram $p_{i}\left(\left[Que\right],\left[Ca\right]\right)$ of values in the range $\left(0,10\sigma_{P}\right)$ with K=1000 bins (spaced by $0.01\sigma_{P}$). Using this histogram, find the entropy associated with a given frequency amplitude of the power spectrum (variability of a single peak across the subsequent time windows) as follows:

(11) $$H_{i}\left(\left[Que\right],\left[Ca\right]\right)=-\sum_{k=1}^{K}p_{i}\left(\left[Que\right],\left[Ca\right],k\right)log_{2}\left(p_{i}\left(\left[Que\right],\left[Ca\right],k\right)\right)$$

### 3.3. Empirical Mode Decomposition

Empirical Mode Decomposition (EMD) is a data-driven technique that decomposes a signal X(t) into a finite set of components known as intrinsic mode functions (IMFs) and a residual signal $r_{n}\left(t\right)$:

(12) $$X\left(t\right)=\sum_{i=1}^{n}C_{i}\left(t\right)+r_{n}\left(t\right),$$

where $C_{i}\left(t\right)$ denotes the *i*-th IMF, and $r_{n}\left(t\right)$ represents the final residual.

Each IMF captures locally dominant oscillatory modes present in the data. A key feature of EMD is its ability to effectively analyze nonlinear and non-stationary time series, unlike classical techniques such as the Fourier Transform. This adaptability stems from the empirical, data-driven nature of EMD: the decomposition is entirely determined by the intrinsic characteristics of the signal without requiring any predetermined basis functions.

The method was originally introduced by Huang et al. in 1998 [63]. The standard EMD procedure consists of the following steps:

1. Identify all local maxima and minima of the signal.
2. Construct the upper and lower envelopes by interpolating between the extrema using cubic spline interpolation.
3. Compute the mean envelope m(t) as the average of the upper and lower envelopes. Subtract this mean from the original signal to obtain a candidate function h(t)=X(t)−m(t).
4. Verify whether h(t) satisfies the two necessary conditions for an IMF:
  - The number of zero crossings and extrema differ at most by one.
  - The local mean, defined by the average of the upper and lower envelopes, is approximately zero at every point.
5. If the conditions are not met, treat h(t) as a new input and repeat steps 1–4 (the so-called ‘sifting process’).
6. Once an IMF is identified, subtract it from the original signal and apply the same procedure to the resulting residual. The decomposition terminates when the residual satisfies the following stopping criterion:
  - The residual contains at most one extremum (either a maximum or minimum), or is a monotonic or constant function, which typically represents the trend of the signal.

In this work, we divided the available patch-clamp data into the non-overlapping subseries of *n* = 100,000 points each. We normalized the subseries using the min-max method to minimize the effects of the original baseline location on the IMFs amplitude. For each resulting normalized subseries, we performed the EMD.

The IMF’s energy is calculated as ${\left(I/Imax\right)}^{2}$, which corresponds to the sum of the squares of the normalized IMF amplitude values rescaled by the subseries length *n*. Thus, the ${\left(I/Imax\right)}^{2}$ describes the time average, amplitude-weighted, relative contribution of a given IMF to the power of the decomposed signal in a unit of time Δt = 100 μs (i.e., 1 over the sampling rate of 10 kHz). Such ’energy’ can be interpreted in terms of the weight of a given mode (a particular IMF) in representing the overall work conducted over potassium ions transported through the investigated channel within Δt.

We analyzed the whole population of IMFs found for all available data at a given concentration of calcium ions and quercetin. The results are presented in the form of 2D histograms of the IMFs’ frequencies and energies.

**Figure 1:**
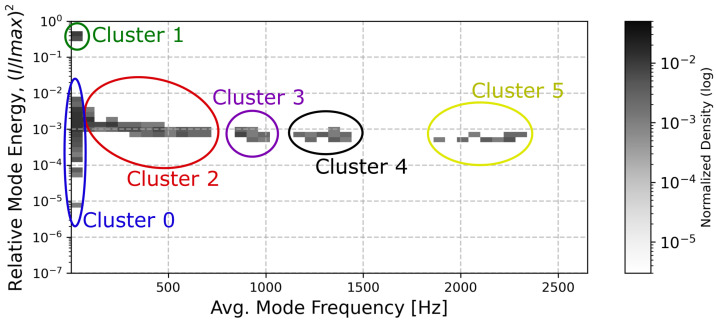
An example of clustering of the EMD results in the form of 2D histograms of IMFs frequencies vs. energies. The clusters are encircled using different colors. The plot was constructed based on the data describing BK channel activity at [Que] = 10 μM and [Ca^2+^] = 100 μM.

The analysis of the signal modes revealed that the IMFs form clusters at specific frequencies and energies. In most cases, six clearly visible clusters are present in the 2D histograms of IFM frequencies and energies. To obtain a clear and reduced representation of the EMD results, we divided the IFMs into six clusters using the K-means algorithm for each histogram (of frequency and ${\left(I/Imax\right)}^{2}$) corresponding to a given combination of [Que] and [Ca^2+^] (Figure 1). Then, for each cluster, we evaluated a weighted average to represent a typical frequency and energy of the IMFs grouped in a given cluster (centroids), relative occupancy of a cluster (defined as the fraction of all IMFs falling into this cluster), and Shannon entropy, which describes the irregularity of the frequency and energy amplitudes of the cluster’s elements.

## 4. Results

### 4.1. Standard Measures: Open State Probability and Dwell-Time Distributions

**Figure 2:**
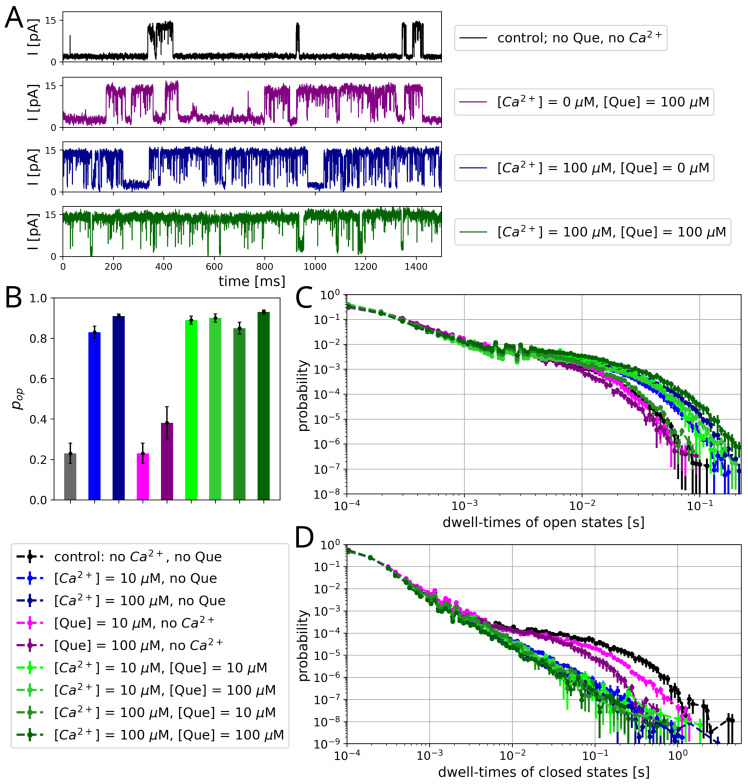
The representative exemplary samples of the time series of single-channel currents recorded at different concentrations of calcium ions and quercetin at *U* = +50 [mV] (**A**). The open state probability values ($p_{op}$) obtained at different combinations of calcium ions and quercetin concentrations are represented by different colors in the bar plot (**B**). Panels (**C**,**D**) show the dwell-time distributions of the open and closed BK channel states, respectively.

The open state probabilities obtained from the experimental data confirm the channel-activating effects exerted by both analyzed agents, Que and Ca^2+^ (Table 1, Figure 2). Notably, the applied concentrations cover the full operational range at which these substances are capable of activating BK channels. The range of Ca^2+^ concentration that typically effectively activates the BK channels is 1–100 μM [36] and the range of quercetin concentration that effectively activates the BK channels is 0.1–100 μM [37]. Therefore, the maximal open-reinforcing effect of a given channel modulator is expected at 100 μM of either substance. As could be anticipated, the obtained increments of $p_{op}$s clearly demonstrate that Ca^2+^ is a stronger channel-activator than Que (Table 1 and Figure 2). Moreover, no clear additional $p_{op}$ increase is observed with the addition of quercetin at full Ca^2+^-related channel activation (i.e., at [Ca^2+^] = 100 μM). There is even a slight decrease in $p_{op}$ at [Que] = 10 μM and [Ca^2+^] = 100 μM when compared to the $p_{op}$ obtained in the absence of Que at the same calcium concentration. Conversely, increasing the Ca^2+^ concentration at a fixed [Que] = 100 μM results in a significant increase in $p_{op}$ from 0.38 ± 0.08 (no calcium ions) to 0.93 ± 0.01 (at 100 μM Ca^2+^).

The dwell-time distributions of open states are double-exponential regardless of the calcium and quercetin concentrations (Figure 2C). One can, however, observe the more frequent occurrence of relatively long-lasting openings in terms of the high availability of Ca^2+^ ions compared to the data obtained in the absence of Ca^2+^ at any quercetin concentration. The only exception is the open dwell-time distribution obtained at [Ca^2+^] = 100 μM and [Que] = 10 μM, which differs only slightly from the control. When quercetin is administered in the absence of calcium, a slight change in the open dwell-time distribution can be seen (with a low stabilization of intermediate-length states).

The discrepancies between Ca^2+^- and Que-related BK channel activation are even more pronounced at the level of the dwell-time distribution of closed states, which takes the form of power-law scaling for calcium activation and a multi-exponential curve (double/triple-exponential) for control and sole quercetin activation (Figure 2D). The multi-exponential distribution can be interpreted in terms of the existence of several stable conformational states of a given type (open/closed) that differ notably in their characteristic lifetime [9,24]. Therefore, one anticipates 2–3 discernible closed states in the control or sole-Que case. On the contrary, power-law scaling (found in the dwell-time distribution of closed states in cases where Ca^2+^ ions were present in the solution) suggests that there is no characteristic timescale, which is typical for a complex manifold of conformational states, sometimes interpreted as fractal in nature [64,65,66]. Again, in terms of sole quercetin activation, a slight increase in the probability of intermediate-length states occurrence can be observed (Figure 2D). Interestingly, in the absence of calcium, channel stimulation by quercetin in a concentration of 10 μM does not lead to a change in the open state probability (Table 1). Nonetheless, it affects the distribution of open and closed channel states.

### 4.2. Results of the Entropic Analysis

#### 4.2.1. Shannon Entropy of the Dwell Times

**Figure 3:**
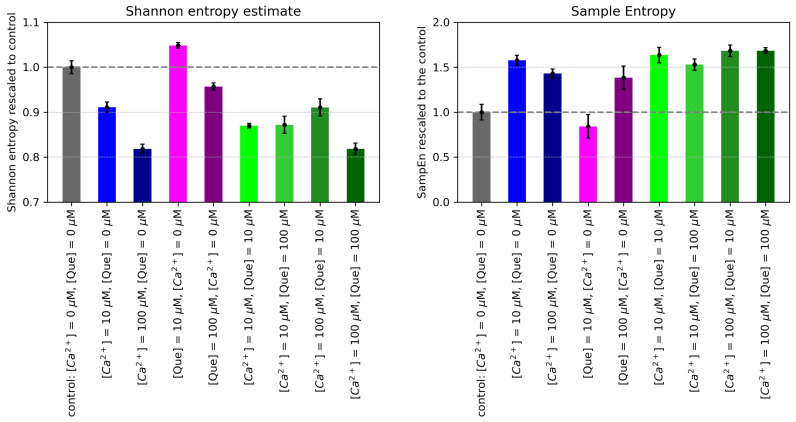
Shannon entropy estimate evaluated for the dwell-times of channel states based on their normalized histogram. The presented values are presented as mean values ± standard error calculated for all available dwell-time series at fixed at concentrations of Ca^2+^ and Que, and rescaled to control (**left panel**). Sample entropy (SampEn) obtained for the dwell-time series at different concentrations of Ca^2+^ and Que. SampEns are presented as mean values ± standard error calculated for the available dwell-time series’ windows of 4000 points length at fixed concentration of both channel-activating agents.

Visual inspection of the dwell-time distributions allows one to identify the main qualitative changes that occur as a result of Que- and/or Ca^2+^-related channel activation. To describe the changes in the overall dwell-time distribution irregularity using a single and well-defined number, the Shannon entropy was used. The Shannon entropy estimates based on the normalized dwell-time histograms obtained at different Que and/or Ca^2+^ concentrations are presented in Figure 3, in the left panel. The results indicate the dose-dependent decrease in dwell-time Shannon entropy in the case of Ca^2+^-activation in the absence of quercetin. This, obviously, reflects the tendency of the dwell-time histogram to stabilize with long open states without intermediate-length openings that otherwise increase entropy. Also, within the closed states, the occurrence of long closings becomes unlikely and ceases to contribute to the entropy. Ultimately, the dwell-time distribution tends toward a sharp peak for short closings and a peak for long openings, without any contributions in between. In the case of quercetin activation in the absence of Ca^2+^, the Shannon entropy increases for [Que] = 10 μM. This likely happens because it is a less potent channel activator than Ca^2+^ and the open states are not yet stabilized at this Que concentration, as the closed states were stabilized for the control. We end up with a broad distribution of dwell times in the histogram because of the changes in the open and closed dwell-times’ structure, e.g., the stabilization of intermediate-length closed states $10^{-3}$–$10^{-2}$ s and only slightly lower occurrence of the relatively long-lasting closures. When the [Que] reaches 100 μM, Shannon entropy decreases in relation to control (due to the leading effect of lowering the probability of long-lasting closed states).

The decrease in the Shannon entropy of the dwell-times due to open-state stabilization is very pronounced in the case of Ca^2+^-activation—either alone or in combination with Que. It is worth mentioning that for Que mixtures with [Ca^2+^] = 10 μM, Shannon entropy shows similar values; nonetheless, these lower than those for the pure [Ca^2+^] = 10 μM (this could imply a synergy in open-state stabilization). In turn, at [Ca^2+^] = 100 μM, one can easily discern a relative increase in the Shannon entropy for [Que] = 10 μM in comparison with [Que] = 0 μM or [Que] = 100 μM (so quercetin at low concentrations may interfere with calcium activation).

#### 4.2.2. Sample Entropy in the Analysis of Dwell Times

The SampEn values obtained for the dwell-time series at different calcium and quercetin concentrations are presented in Figure 3, in the right panel. Before commenting on these results, let us recall that SampEn is especially sensitive to the presence of repetitive sequences within the analyzed signal. SampEn provides a numerical value that characterizes the predictability of the analyzed series. A lower SampEn indicates higher regularity. A higher SampEn suggests more randomness in the data structure [58,59]. In the Methods section, we related the SampEn of the dwell times to the connectivity of the Markov chain graph of an ion channel. To make use of this observation, following the literature, in the analyzed case of the BK channel dynamics, we can assume that there are two functional open (O_1_, O_2_) states of discernible average length, and three closed states (C_1_, C_2_, C_3_) of discernible average length [24]. If the action of a given factor leads to an increase in SampEn, this likely means that the total number of states (O_*i*_ and/or C_*j*_) and/or paths linking open and closed states (O_*i*_ and C_*j*_) that are attainable from the energetic and spatial point of view has increased. Since we calculated SampEn for the embedding dimension equal to 2, it describes the complexity of possible transitions between open–closed and closed–open states of a certain length. Our results show that BK channel activation by calcium ions and quercetin generally increases the complexity of pairs of subsequent dwell times of channel states, which suggests the larger kinetic diversity in channel gating (Figure 3, in the right panel). For example, pure calcium activation follows the expected course of low entropy for zero calcium (stable closed states), maximum entropy for partial calcium stimulation (switching), and a drop in entropy for maximum calcium stimulation (open states become stable). The only exception is the SampEn obtained for the data at [Que] = 10 μM and [Ca^2+^] = 0 μM, where it takes a lower value than the control. A phenomenon like this may result after a weak activation, for example, through the elimination of short spike openings in favor of longer openings or by the fragmentation of long closings by spike openings (of limited effect on $p_{open}$) that occur more frequently. In the first case, the number of open conformation targets increases, while in the second case, the number of target closed states from the open spike increases.

An extended explanation of the Shannon entropy and sample entropy (SampEn) analyses of the dwell-time series is provided in Appendix A.

### 4.3. Results of the Spectral Analysis

#### 4.3.1. The Power Spectrum and the Relative Power Spectrum

**Figure 4:**
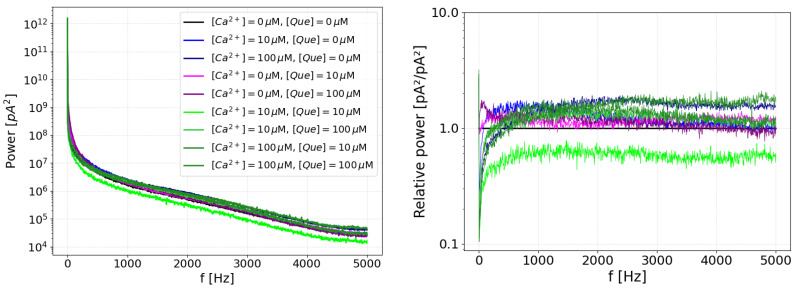
Power spectrum of single-channel currents describing the BK channel activity at different combinations of [Ca^2+^] and [Que], showing both their individual and simultaneous effects. The membrane potential was fixed during the patch-clamp recording at *U* = +50 [mV].

The spectral analysis of the activation effect of Ca^2+^ and quercetin was first performed by calculating the median of the power spectrum of the current signal, built from the power spectra of all available recordings under particular stimulation. The results revealed many differences, which are depicted in raw form in Figure 4, in the left panel.

**Figure 5:**
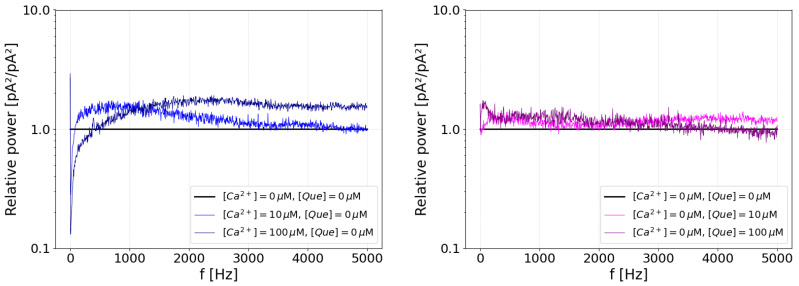
Power spectrum of single-channel currents describing the BK channel activation by a single agent, i.e., calcium ions (**left panel**) and quercetin (**right panel**). The membrane potential was fixed during the patch-clamp recording at *U* = +50 [mV].

The frequency shape of the power spectra generally follow the characteristic of the 1/f noise, which is of heterogeneous origin and may be attributed to various sources; for example, to the effect of the pipette and holder [9] or to the fractal signal properties, like intermittency [67], or to general conformational fluctuations in the channel protein [68]. This general characteristic is not of interest to us, and we would rather aim to find some fingerprint deviations from this basic power spectrum shape. To achieve this, and obtain some information regarding the response to applied stimuli, we replotted the data with respect to the power spectrum of the unstimulated channel $\hat{P}\left(\left[Que\right]=0,\left[Ca\right]=0,f\right)$ (control data), and directly observed the ratio by which the peaks grow or decrease in response to the activating agent, i.e., calcium ions or quercetin. This method removes the baseline variability while retaining information about relative changes in the spectrum. The plot of activations resulting from the simultaneous action of quercetin and calcium is shown in the right panel of Figure 4. For reference, Figure 5 shows the relative power spectrum plots for activations by pure calcium or pure quercetin.

It can be seen in these plots that weak calcium activation increases the low-frequency components (i.e., an open-channel current as a constant component instead of a closed-channel current). Increased calcium activation lifts the high-frequency tail of the power spectrum (to a level unattainable by Que activation), also affecting the low-frequency component. This interaction may occur because high-frequency, rapid switching reduces long open intervals, destabilizing the low-frequency peak.

In contrast, the effects of quercetin on the power spectrum are minor: there is a transient effect for low quercetin concentrations on the high-frequency tail (destabilization of closed states), which vanishes at larger quercetin concentrations in favor of an increase in low frequencies (stable open states). In case of simultaneous activation by both agents, for large calcium stimulation, large quercetin stimulation has little effect, and the activation looks similar to that imposed by calcium alone. Interestingly, a moderate amount of quercetin changes the power spectrum. This is also in accordance with the power spectra for pure components, where a moderate amount of quercetin has the capacity to influence the large-frequency tail of the current, interfering with the calcium effect and effectively changing the shape of the high-calcium power spectrum into that of the low-calcium activation spectrum. The biggest change to the power spectrum, i.e., a large drop in power spectrum values in the whole considered range, occurs for low calcium and low quercetin activation ([Ca^2+^] = 10 μM, [Que] = 10 μM). Such effects are absent in either pure activation by calcium or pure activation by quercetin and must be attributed to their cooperation.

It follows from the above that calcium activation dominates; low amounts of quercetin have the potential to compete with calcium ions but high amounts do not. A reasonable conclusion would be that high amounts of quercetin in the presence of calcium ions undergo some sort of self-inactivation (but calcium ions are required for that, since otherwise this quercetin inactivation is not observed).

#### 4.3.2. Entropy of the Power Spectrum Peaks

**Figure 6:**
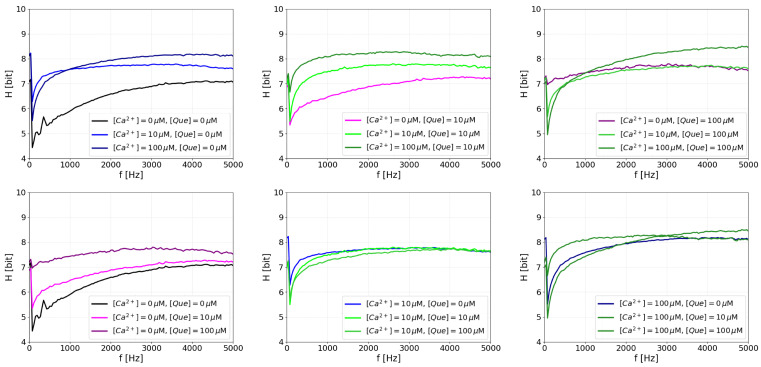
The entropies of the power spectrum peaks in varying [Que] and [Ca^2+^] activation.

Because a lot of information may be contained in the frequency domain, we also analyzed the Que- and Ca^2+^-related entropy associated with a given frequency amplitude of the power spectrum $H_{i}\left(\left[Que\right],\left[Ca\right]\right)$. In other words, we analyzed the variability of individual power spectrum peaks for prescribed frequencies as they evolve in subsequent time windows. The results are presented in Figure 6. For pure activating agents, changes in the entropy of the low-frequency peak are as follows: for calcium activation, the entropy increases (because the channel turns from the off state to a switching state), and then drops again (as the open state becomes stable and switching activity decreases). For quercetin activation, however, the situation is different. Since this is not as potent an activator as Ca^2+^ ions, the low-frequency part grows monotonically with quercetin content (it does not reach the activation level at which the open state becomes stable).

What makes the activations by quercetin and calcium different, from the perspective of the entropy of spectral peaks, is the mid-frequency range. The entropy for this range grows rapidly to saturation with even small amounts of calcium, but the effect for quercetin activation is much more gradual. We could say Ca^2+^ promotes the destabilization of closed states at all frequencies, while quercetin is less potent in fast switching.

Introducing large quercetin levels at either low or high calcium levels has a minor effect on the entropy plots. Reflecting the findings of the basic spectral analysis, introducing small amounts of quercetin changes the high calcium plot by enlarging the entropy of the low-frequency components (i.e., there may be some entropy-related switching). This finding again highlights the fact that low amounts of quercetin have the potency to compete with calcium activation, whereas high levels of quercetin eliminate this interaction.

### 4.4. Results of the Empirical Mode Decomposition

**Figure 7:**
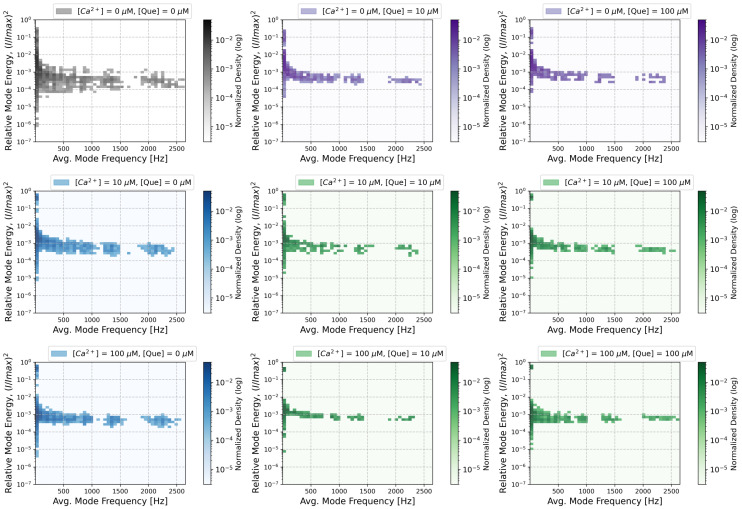
Normalized 2D histograms of IMFs energies (presented as ${\left(I/Imax\right)}^{2}$) and frequencies obtained by the EMD of normalized patch-clamp signals at varying [Que] and [Ca^2+^].

The bi-dimensional histograms of IMFs frequencies and the corresponding energies, expressed as normalized squared current amplitudes obtained at different calcium and quercetin concentrations, are presented in Figure 7. Each subplot presents how the applied [Ca^2+^] and [Que] combination affects the locations of the IMFs representing the component oscillatory modes of the analyzed signals group in the frequency and energy domains.

A visual inspection of the results presented in Figure 7 suggests that the control data exhibited the greatest diversity of IMFs in terms of both frequency and energy. In these circumstances, gating fluctuations appear to be the least restricted. Administering either channel-activating agent causes the oscillatory components of the signal to focus within the energy axis. This means that the signal displayed similar characteristics in all analyzed windows (of 100,000 samples, equivalent to 10 s), i.e., it did not switch between states that would display characteristics that cannot average out within this window size. The results of the Shannon entropy of the dwell-time distribution largely support this effect (cf. Figure 3, the left panel). The evident grouping of the results in (frequency, energy) space inspired us to divide them into six non-overlapping clusters.

**Figure 8:**
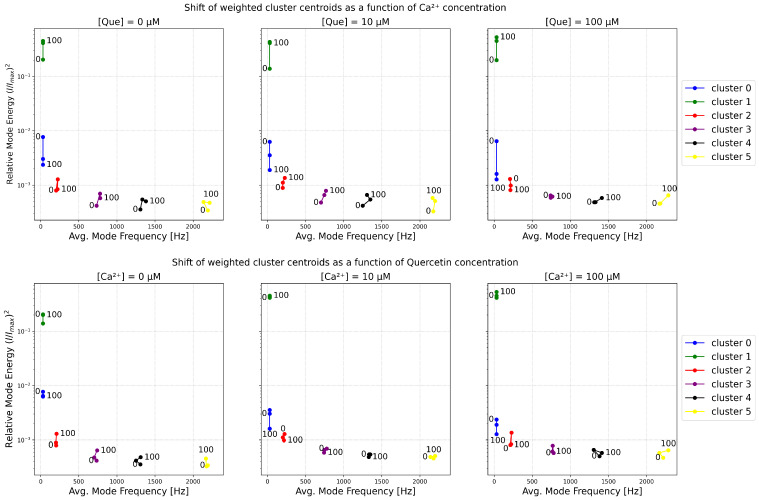
The changes in the weighted average of IMFs energies (normalized squared amplitudes) and frequencies corresponding to six distinguished clusters for different concentration of calcium ions and quercetin.

The structure of the IMFs’ energies and occupancies corresponding to the first frequency bin (below ∼60 Hz) is of particular importance, as these clearly form two distinct clusters that differ significantly in terms of signal amplitude and dispersion. The high-energy cluster (Cluster 1) ought to correspond to long-lasting open states—sustained channel activity. In contrast, the low-energy cluster (Cluster 0) mostly corresponds to the stable long-lasting closed states. However, it may also describe the destabilization of some rare, relatively long open states due to the simplicity of the EMD algorithm and the averaging implemented. This makes the algorithm not very sensitive to the type of amplitude variation, such as stabilization of the baseline or stabilization of the signal near its maximal amplitude. For low activation, Cluster 0 dominantly describes the closed state with minor excursions to openings, hence the broad distribution. As activation increases, a stable open state separates from the wide distribution, and this state is not likely to be disturbed by closings (it separates from the low-conduction cluster). These effects are visible in Figure 8, where the increase in [Ca^2+^] results in an increase in the distance between Clusters 0 and 1 along the ${\left(I/Imax\right)}^{2}$ axis.

**Figure 9:**
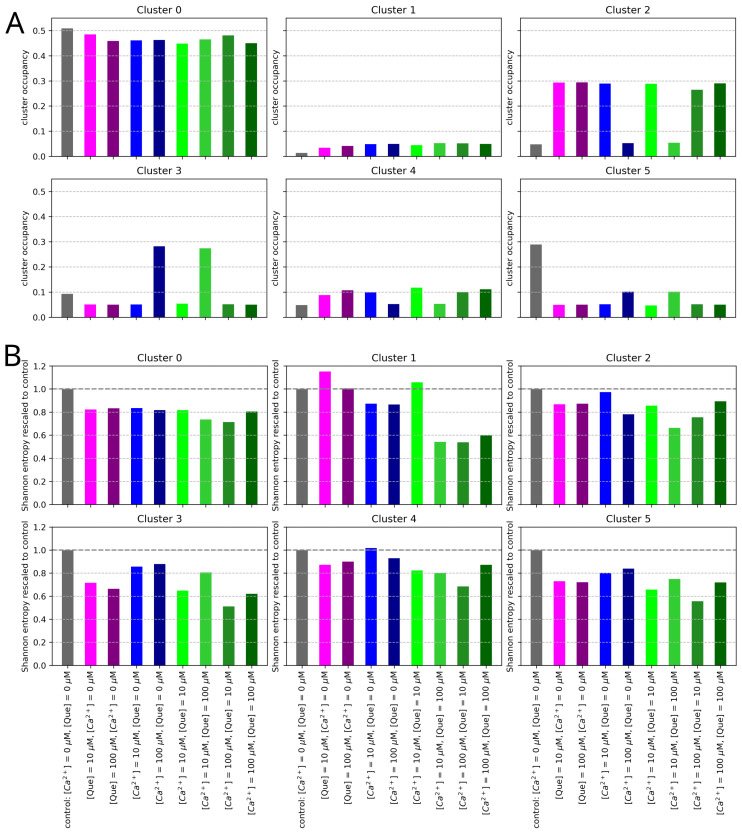
The changes in IMFs clusters’ occupancy (the fraction of IMFs falling in a given cluster to the overall number of IFMs) at varying [Que] and [Ca^2+^] (**A**). The changes in the Shannon entropy of the frequency and energy distribution for the distinguished clusters obtained at different calcium and quercetin concentrations (**B**).

At higher frequencies, four other clusters are visible (Clusters 2–5). These clusters are particularly distinctive when mixtures of calcium ions and quercetin are administered. The changes in the weighted average of clusters’ energies and frequencies (centroids) are presented in Figure 8, while their occupancy and corresponding Shannon entropy values are presented in Figure 9. As can be seen, pure Ca^2+^ activation, in many cases, transiently increases the energy of intermediate frequencies ([Ca^2+^] = 10 μM) as the closed state destabilizes and switching (at characteristic dwell time durations) occurs. Then, at [Ca^2+^] = 100 μM, the energy of these modes decreases as the open states become stable and oscillations disappear. This is different for quercetin activation, where the channel does not appear to reach a stable open state but instead exhibits persistent open–closed flickering. In agreement with the results of spectral analysis, one can observe a large effect of Ca^2+^ on the high-frequency tail in comparison to Que.

A decrease in the occupancy of Cluster 0 reflects a reduced proportion of time that the system spends in long-lasting states associated with low conductivity. An increase in ${\left(I/Imax\right)}^{2}$ and occupancy of Cluster 1 (well-pronounced at calcium activation, Figure 9, in the upper panel) represents an increase in high-amplitude, low-frequency patch-clamp signal components corresponding mostly to stable channel open state. These shifts align with an increased open-state probability of the channel in the presence of channel-activating agents. A greater tendency of the channel to undergo a transition into relatively stable open conformations translates to a relatively high signal amplitude due to the enhanced ionic transport capabilities.

Clusters 2–5 represent components in higher frequency ranges. Notably, an increased occupancy of Cluster 2 (frequency ∼200–250 Hz) and/or Cluster 3 (frequency ∼700–800 Hz) is observed in the presence of calcium ions and/or quercetin, at the expense of Cluster 5 occupancy (frequency of over ∼2000 Hz). This probably corresponds to the loss of the isolated opening spikes (Cluster 5) in favor of longer open states (bursts of openings) for the stimulated channel in relation to control signals. When calcium is administered at a high concentration, either in the absence of quercetin or when [Que] = 100 μM, components of relatively high frequency ∼700–800 Hz (representing relatively fast open-closed/closed-open switching) frequently occur in signals. In contrast, solely quercetin-related channel activation, low calcium activation (regardless of the Que presence), or combined treatments ([Que] = 100 μM + [Ca^2+^] = 10 μM or [Que] = 100 μM + [Ca^2+^] = 100 μM) favor slower signal components (∼200–250 Hz). This suggests that in the complex gating dynamics of the BK channel, there are some relatively fast calcium-dependent components (corresponding to a few millisecond-long dwell times of channel states), which can be mitigated by the presence of quercetin (resulting in the prolongation of these dwell times to several milliseconds). The plot of Cluster 5 occupancy suggests that modulator binding appears to reduce the number of isolated transient opening events. The overall reduction in the irregularity of amplitude and frequency distributions within recognized clusters, presented in the form of a Shannon entropy decrease in Figure 9B, may indicate the suppression of random activity and more stable patterns of channel gating in the presence of Ca^2+^ and/or Que.

## 5. Discussion

### 5.1. General Remarks on Calcium–Quercetin Interactions

In this work, we investigate how the interplay between quercetin and intracellular calcium concentration shapes the gating behavior of BK channels. The activity of BK channels can be modulated by many stimuli (e.g., Mg^2+^, CO, Omega-3, ethanol), as well as the generic activating effects of elevation of the cytoplasmic [Ca^2+^] and membrane depolarization [44]. Among the BK channel modulators, quercetin can be considered a potent activator according to the dedicated studies on different BK channel variants, i.e., BK channels in the human bladder carcinoma cell line [38], coronary smooth muscle cells [69], murine smooth muscles (ileal myocytes) using liposomal quercetin [39], mitochondrial BK (mitoBK) channels from human endothelial cells EA.hy926 [40], and mitoBK in human bronchial epithelial cells (HBE) [42]. Here, we demonstrate that BK channels from the cell membrane of the HBE cells are no exception, and quercetin can increase their open-state probability, particularly when administered at high concentrations. At lower levels, quercetin can alter their gating kinetics and compete with Ca^2+^ ions.

Calcium ions are one of the key signaling agents in biology. They are also generic activators of BK channels. When anchored to the BK channel’s cytoplasmic gating ring, Ca^2+^ ions lead to expansion of this domain and, further, to conformational changes across all domains of the channel. These structural rearrangements finally result in the tendency of the channel to stay in its functionally open states [44,45,46,47,48]. Much more is known about voltage- and Ca^2+^-sensor coupling [46] in BK channels. The three main functional domains, i.e., voltage sensors, pore-gate domain, and Ca^2+^-sensors, operate independently but are allosterically coupled. Consequently, BK channels can be activated by either voltage or Ca^2+^. However, the activation of one sensor type triggers conformational rearrangements in the other sensors and the gate, shifting the probability of the open state even more effectively. This phenomenon is known as functional cooperativity between the channel domains. Whether weaker biochemical activators, like quercetin, can couple with voltage or Ca^2+^ sensors in BK channels is still puzzling. If such coupling between the Que-binding site(s) and the Ca^2+^-sensor is possible, it is definitely quite subtle.

Our results confirm the thesis formulated above. They indicate that there is a complex and nuanced way in which Ca^2+^- and Que-binding can couple, which has to affect the sensors–pore-gate interactions and, consequently, define the actual probability distribution of BK channel conformations, the dynamics of conformational switching, and, consequently, the functional effectiveness of the K^+^ transport.

Considering the quercetin interactions with the Ca^2+^-bound and Ca^2+^-free BK channel structures, in both cases, such interactions seem to be mechanistically possible, and multiple Que-binding sites should exist (similarly to the existence of several binding sites for the other, relatively small, amphiphilic channel-modulating molecules, like NS11021 [70]). The nature, location, and binding energy between a particular binding site and Que molecule should depend on whether BK channel is in its Ca^2+^-bound or Ca^2+^-free state, or in the multiple intermediate states, that could be reached at medium levels of [Ca^2+^] (≃ micromoles up to 100 μM), where each Ca^2+^ sensor cannot be ‘saturated’ by Ca^2+^ binding. In the presence of Ca^2+^ ions, the accessibility of the BK channel (both its gating ring and channel pore) to quercetin molecules seems to be evident due to the strong tendency of PG and GR to be wide open. In turn, in the nominal absence of calcium ions, GR and the intracellular gate are narrow. Nonetheless, even in the Ca^2+^-free BK channel structure, the pore remains wide enough to allow large molecules to pass through or access the inner water-filled cavity inside the pore [46]. Moreover, in the Ca^2+^-unbound BK channel structure, molecules with relatively large hydrophobic regions can also access the inner cavity via membrane-facing fenestrations [46,71]. According to our results, quercetin can be coordinated with the Ca^2+^-unbound BK structure, and this effectively influences the open/closed kinetics. When Que is administered at a low concentration ([Que] = 10 μM at [Ca^2+^] = 0 μM), the gating kinetics is altered without significant changes in $p_{op}$. In turn, when Que is administered at high levels ([Que] = 100 μM at [Ca^2+^] = 0 μM), this leads to considerable perturbations in gating dynamics and equilibrium. At these terms, one can observe a Que-related increase in open state probability from $p_{op}$ = 0.23 ± 0.05 (control) to 0.38 ± 0.08 at full Que-activation.

For mixtures of Ca^2+^ and Que, there is an equivocal situation from the perspective of relatively high channel activation, recognized by the high $p_{op}$ values at either Que concentration in the presence of Ca^2+^ ions. Nevertheless, more exhaustive kinetic inspections of the $p_{op}$ and dwell-time distributions of open/closed BK channel states reveal some interesting effects in this regard. Namely, full channel activation ($p_{op}$ > 0.90) is reached at high [Ca^2+^] (no Que) or when a mixture of Ca^2+^ and Que is administered. There is, however, one exception at [Ca^2+^] = 100 μM and [Que] = 10 μM, for which $p_{op}$ slightly decreases in comparison with [Ca^2+^] = 100 μM and [Que] = 0 μM. The next observation is that channel activation by Ca^2+^ (regardless of the presence of quercetin) results in a power-law-like distribution of closed states, suggesting that there is no characteristic dwell-time scale for non-conducting states of the Ca^2+^-bound channel.

The distributions of the dwell times of channel states are continuous, and it is difficult to precisely identify the characteristic, possibly narrow time intervals in which the differences between the analyzed data groups corresponding to different combinations of [Ca^2+^] and [Que] are the greatest. Thus, in this article, we applied entropy-based, spectral, and EMD-based methodologies to inspect the changes in BK channel gating dynamics in more detail. These methods allowed us to indicate the ranges in the frequency domain in which the effects of the coupling of the applied agents—Ca^2+^ and Que—are well pronounced. Our results suggest that there may be more spatial and energetic constraints within the manifold of available open/closed conformations in the presence of Ca^2+^/Que in comparison with the unbound BK channel structure (control): there are well-localized clusters in the frequency and ${\left(I/Imax\right)}^{2}$ domains (narrower distributions in Figure 7 and a decrease in clusters’ Shannon entropy in Figure 9), the stabilization of long-lasting openings by calcium coordination (Figure 6, Figure 8 and Figure 9), and some interesting effects exerted by Que at intermediate-frequency scales (clearly shown through the changes in cluster occupancy in Figure 9).

In light of the obtained results, the effects of the functional coupling of Ca^2+^- and Que-binding sites depend on the Ca^2+^ and Que concentration, which suggests quite original Que/Ca^2+^ competitive traits at a low Que concentration and symptoms of Que ’deactivation’ at [Que] = 100 μM in the presence of calcium ions. The complexity of Que-Ca interdependency may stem from the multifaceted mechanism of Que action. What is known from the literature is that quercetin activates the BK channel by binding inside the pore [41]. Nonetheless, its presumptive binding in the gating ring, where it could strongly compete with Ca^2+^, cannot be ruled out, as MD docking in [41] was only performed within the Paxilin-binding sites located within the membrane-spanning pore-gate domain, and the gating ring was omitted in that study. Moreover, quercetin can also affect the BK channel through the membrane [50], where it can significantly affect membrane-mediated cell signaling cascades through alterations in lipid packaging, fluidity, and permeability [51,52,53]. These quercetin–membrane interactions can indirectly affect the functioning of integral membrane proteins (including ion channels) and modulate their structure and function [72,73,74,75].

It is worth noting that the availability of Ca^2+^ and, consequently, its ability to bind to the BK channel structure strongly affect the outcomes of quercetin-related effects on channel activation and gating. However, the modulatory effects of quercetin and calcium on BK channels may differ quantitatively and functionally in different cell types and channel locations (e.g., in plasma membrane or inner mitochondrial membrane) due to the specific molecular composition of particular channel isoforms, the coordination with different regulatory subunits (β1–4, γ1–4), and post-translational modifications aimed at adaptation of the channel structure to the existing cellular signaling pathways. Additionally, the variability in the lipid and protein composition of different membranes may also contribute to possible quantitative differences in the flavonoid-mediated activation levels [53]. In excitable cells, like neurons, cardiac myocytes, or endocrine cells, where channel subunit composition is highly specialized and tightly linked to calcium-dependent signaling cascades, the presence of quercetin may lead to amplified (or qualitatively distinct) responses.

### 5.2. The Interpretative Potential of the Entropy-Based Metrics

The results of the entropic analysis, supported by some kinetic inspections and the available contemporary literature on mechanistic principles of BK channel gating [46,70,71,76,77,78], can be utilized in the formulation of running hypotheses about the molecular mechanism of Que- or/and Ca^2+^-related changes in the BK channel functioning, which can be later verified by MD docking and experimental studies using appropriately designed channel mutants.

#### 5.2.1. Effects of Ca^2+^

**Figure 10:**
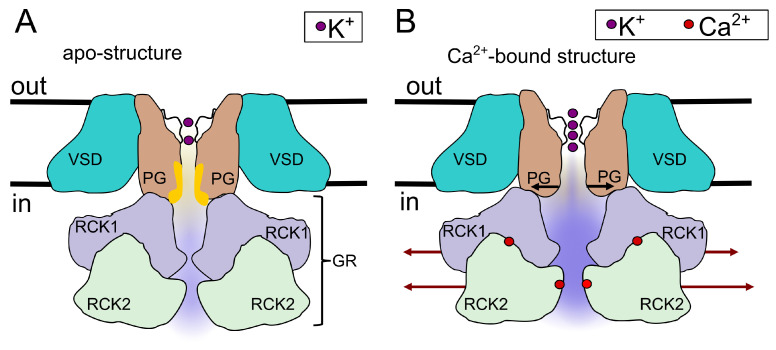
The schematic representation of the BK channel structure in its Ca^2+^-free (apo-structure) (**A**) and Ca^2+^-bound (**B**) states. In the Ca^2+^-free structure, the inner cavity is relatively narrow, both within the pore-gate domain (PG) and the gating ring (GR) formed by RCK1 and RCK2 domains. In the apo-structure, lipids (depicted in yellow) can face the inner pore through the fenestrations. This structure (**A**) facilitates pore dewetting (functionally closed states). In the Ca^2+^-bound structure, the PG and GR are relatively wide, which supports ’wet’, functionally open states. The intensity of the shades of blue represents the relative accessibility of water molecules to the channel pore. VSD denotes the voltage-sensing domain.

First, let us refer to the relatively well-understood mechanism of Ca^2+^-activation of the BK channel in the absence of quercetin and consider the corresponding changes in Shannon entropy. In the Ca^2+^-free structure (also called the Ca^2+^-unbound or apo-structure), the key functional domains and regions support the frequent occurrence of a closed macrostate, as presented in Figure 10. This macrostate pertains to the channel conformations when its hydrophobic pore significantly narrows (pore diameter decreases below 10 Å and it should undergo dewetting, which poses a significant free energy barrier to ionic transport and impedes the permeation of hydrated K^+^ ions [76,79]). In the apo-structure, the channel pore-gate domain, as well as the large cytoplasmic gating ring, are relatively narrow [46], which makes the pore prone to dewetting [76] (frequent and relatively long closed states are observed). Channel fenestrations are filled with lipids that also enhance dewetting [46,71,77]. Open states are not precluded in the apo-structure, but they should mostly last for a relatively short time. In turn, Ca^2+^-binding results in a large-scale structural rearrangement of the channel—the gating ring widens, but also, to a lesser extent, the pore-gate domain [46], fenestrations become occluded, and consequently, there are no lipids facing the inner cavity in this region of the channel [77]. These changes collectively make the pore less prone to dewetting. In the Ca^2+^-bound structure, there an important hydrophobic region remains just below the selectivity filter, and its presence sustains the possibility of rare pore dewetting events—thus, functionally closed states are still possible. Nevertheless, due to the preference of keeping the gating ring and the gate wide open, pore rehydration after rare dewetting events should proceed more easily for the Ca^2+^-bound structure than for the apo-structure.

What kind of observable changes are expected in the patch-clamp signal and the corresponding distributions of the open and closed states after calcium binding? This results in the occurrence of longer open states (wet states) in comparison with the open states of the apo-structure, and the longest wet states should be mostly stabilized (quite well-occupied long tail in the open dwell-time distribution in Figure 2D). Closed states are still allowed since the PG and GR widths constantly fluctuate (thermal movements), and in some rare transient quasi-states, the pore may start to undergo dewetting (after an open state of arbitrary length); this process starts from the hydrophobic region of the pore.

After dewetting in the Ca^2+^-bound conformation, it is not certain whether the whole pore will become dehydrated due to the relatively high influx of water molecules through the wide open GR, which counteracts dewetting. Thus, some current fluctuations in the open-state current amplitude can occur, where, due to the sampling rate of 0.1 ms, some of the short effects can average out with subsequent openings, without reaching the current amplitude of a closed state.

In the case of a full dewetting, there should be a certain minimal closed dwell-time, which is needed to re-hydrate the dry pore (which is beyond the resolution of patch clamp sampling). If a dry state of the pore is reached, the dwell-time of the closed state will depend on the actual fluctuations in the GR’s width and the Brownian dynamics (diffusive fluctuations) of the channel building blocks.

The wider the transient GR state is, the more probable it is that the pore will be rehydrated, and ionic transport will be possible. Since broad GR entry to the pore is preferred, re-wetting of the pore is very plausible in the Ca^2+^-bound state; thus, the number of observable intermediate and long closed states should mostly decline.

With these observations, the expected changes in Shannon entropy with Ca^2+^-activation should relate to a Ca^2+^-related dose-dependent decrease in entropy mainly stemming from the stabilization of open states (preferably intermediate and long states) and destabilization of the shut ones (preferably very short ones close to the edge of the sampling resolution). In this way, Ca^2+^-binding restricts the durations of channel conformational states in comparison to the control, leading to a drop in entropy. These effects are confirmed by our results (Figure 3).

#### 5.2.2. Effects of Quercetin

**Figure 11:**
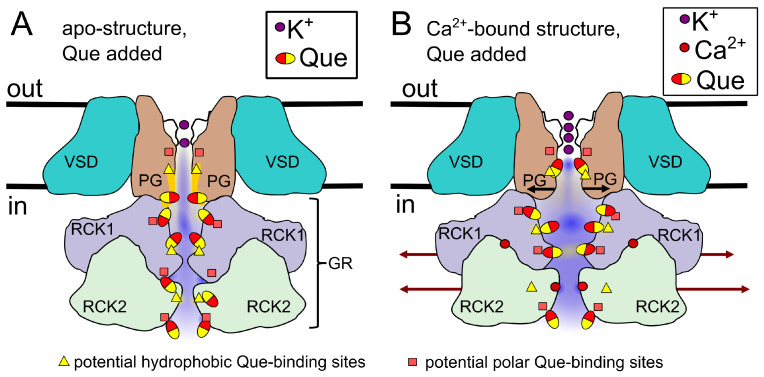
The schematic representation of the possible quercetin-binding effects to the BK channel structure in its Ca^2+^-free (apo-structure) (**A**) and Ca^2+^-bound (**B**) states. In the Ca^2+^-free structure, the inner cavity is relatively narrow both within the pore-gate domain (PG) and the gating ring (GR) formed by RCK1 and RCK2 domains. In the apo-structure, lipids (depicted in yellow) can face the inner pore through the fenestrations and interact with Que hydrophobically. At these terms, Que can expose its polar regions to the channel pore. In the Ca^2+^-bound structure, the PG and GR are relatively wide. The number, type (hydrophobic/hydrophilic), binding geometry, and occupancy of que-binding sides exert complex effects on ’wet’/dewetted states stabilization, which depend on the availability of Que molecules and Ca^2+^ ions. The intensity of the shades of blue represents the relative accessibility of water molecules to the channel pore. The shades of yellow represent hydrophobic regions. VSD denotes the voltage-sensing domain.

Let us now consider what could occur in the presence of quercetin in the case of the Ca^2+^-free structure (Figure 11A). The PG domain should remain relatively narrow; however, it also probably becomes less susceptible to dewetting when compared to the Que-free apo structure. This is because the coordination of quercetin inside the hydrophobic pore regions can decrease their hydrophobic potential (possibly similarly to NS11021 molecules [70]). The quercetin molecule is amphiphilic, and the binding of the hydrophobic part means exposing polar Que regions to the conduction pathway.

The channel’s fenestrations may also play a role in these interactions. The fenestrations are typically filled with lipids that enhance dewetting. However, they also provide options for the amphiphilic Que molecule: it can easily enter the pore through the fenestrations and become anchored within the fenestrations via hydrophobic interactions (as occurred in the other small amphiphilic molecules [70]). In this anchored state, polar Que regions may face the channel cavity (instead of lipids) and become occluded by water, ultimately facilitating open (wet) states.

Quercetin binding to the gating ring may result in small changes in the (otherwise narrow) GR structure and surface charge. Depending on where and how quercetin binds to the gating ring, it can exert the opposite effects, promoting the open state, where Que docks using hydrophobic forces and exposes its polar regions to the ion conduction path, and promoting the closed state, where the Que molecules interact via their polar regions and expose their hydrophobic regions to the ion conduction path.

Considering our results regarding Shannon entropy (Figure 3) and the possibility of counteractive effects of Que, we think that at a low concentration [Que] = 10 μM, [Ca^2+^] = 0 μM, Que molecules may dock preferentially within the BK channel’s gating ring by its polar regions and expose their hydrophobic regions to the ion conduction path (which effectively supports dewetted states). At the same time, quercetin can exert the opposite effects within the channel pore region (binding via hydrophobic interactions, which should support wet states). The complexity of these effects results in an increase in the Shannon entropy of the dwell-time series at [Que] = 10 μM and [Ca^2+^] = 0 μM in comparison to the control. Alternatively, this effect may be simply due to destabilization of the low-entropy closed state, which allows for more open target conformations.

In turn, at [Que] = 100 μM and [Ca^2+^] = 0 μM, i.e., a high availability of Que molecules, the potential binding sites of GR that coordinate Que via hydrophobic regions and expose polar ones to the channel pore are also occupied. Together with the Que effects within the channel pore, this leads to an opening-supportive net effect, lengthening the open states and destabilizing the closed ones. From this reason, Shannon entropy decreases for [Que] = 100 μM and [Ca^2+^] = 0 μM in comparison to control. Alternatively, this may be due to the steady stabilization of the open state with increasing [Que] and the reduction in the shape of the closed dwell-time distribution.

Considering the mechanism of quercetin action in the presence of Ca^2+^ ions (Figure 11B), one could anticipate that the channel’s structural arrangement within PG and GR is mostly predetermined by the Ca^2+^-binding. However, it could be supposed that quercetin may anchor via its non-polar regions to the hydrophobic PG regions. The consequent exposition of the polar Que regions to the ionic transport path should support the open (wet) channel states. More complex interactions might occur within the gating ring. It is important to note that quercetin may dock to different binding sites within the GR in the Ca^2+^-bound BK channel structure than in the case of the apo-structure, with a variety of Que-binding options at intermediate Ca^2+^ levels.

Since the BK channel is a tetrameric protein, where, at a low [Ca^2+^], not all of the channel’s Ca^2+^ sensors can be found in its ligand-bound state, Que may preferentially dock to some binding sites typical of the Ca^2+^-bound or Ca^2+^-free structure. Entropy values can help to determine what can actually happen there. We expect that, at a low Ca^2+^, the net effect of the Que action supports the functional effects exerted by the Ca^2+^ ions. Namely, Que can preferentially bind through hydrophobic forces to the hydrophobic regions of the GR and expose its polar regions to the ionic conduction path, which supports the wet state (recognized as the open state in the signal). Thus, Shannon entropy is lower for the dwell-time series at [Ca^2+^] = 10 μM in the presence of quercetin than at [Ca^2+^] = 10 μM in the absence of quercetin.

At a high [Ca^2+^], all Ca^2+^-sensors are ’saturated’. Therefore, quercetin binds to the binding sites typical of Ca^2+^-bound structure. At [Que] = 10 μM, we observe an untypical increase in the signal’s Shannon entropy. In this situation, Que-binding must result in opposite effects to Ca^2+^-binding. Therefore, we think that quercetin at low levels preferentially binds to the GR of the Ca^2+^-bound channel by its polar regions (hydroxyls and carbonyl oxygen) and exposes its hydrophobic region to the channel axis. These interactions destabilize the channel’s open states and prolong their closed states, which, together with counteracting the open-supporting effects of high [Ca^2+^] in GR and [Que] within the pore region, eventually increase the kinetic complexity of BK channel gating and lead to a relatively high Shannon entropy. At high Que levels, quercetin molecules could also dock in the GR via their hydrophobic binding sites, and expose their polar regions to the channel axis. As a result, the multiple contradictory Que-related effects may partially cancel each other out, and the resulting net effect of both agents (at [Ca^2+^] = 100 μM and [Que] = 100 μM) mostly supports stable open states and counteracts long closed states, which decreases entropy. Alternatively, at high quercetin concentration in the saturating calcium conditions, Que molecules could interact with each other sterically before reaching the polar regions of the pore, canceling the blocking effect in this way.

### 5.3. The Utility of the Entropy-Based, Spectral, and EMD-Related Methodology in the Channel-Oriented Research: Example Relations to Recent Experimental Research

The entropy-based methodology (and the other analytic tools used in this work) can be considered a useful analytic approach to study patch-clamp recordings and the corresponding dwell-time series in a variety of other channel-related problems. Its results have a great interpretative potential in terms of the putative mechanisms underlying channel gating. Below, we present two running examples.

#### 5.3.1. The Ball and Chain Inactivation of the Ion Channels

The entropy-based methodology could be used to address the mechanism of ball-and-chain inactivation, such as BK channel inactivation mediated by *β*2 subunits [78]. According to the mentioned studies [78], the ‘ball’ can bind to multiple sites in the channel’s cavity, where it can actually dock and undock, which eventually results in current fluctuations after inactivation. Therefore, one may expect current conduction patterns in a channel containing the ‘ball’, such as inactivated-open1-inactivated and inactivated-open1-open2-open1-inactivated, where ‘openN’ stands for conformation with the ‘ball’ bound to site ‘N’, which does not completely inactivate the channel. Depending on the number of these sites, their affinities, and the accessibility constraints imposed by the chain length, the entropy will differ.

Entropic results could be compared, for example, to hypothetical experiments where inactivation is carried out by the ‘ball’ with a controlled ‘chain’ length (shortening the ‘chain’). On that basis, one could infer the states excluded from the ‘ball’ dynamics due to the limited chain length.

One could also consider more inactivating sites for the ball than just one. In such a case, one may be interested, for example, in whether the channels always recover from inactivation by the same pathway. In the first case, sample entropy would reveal that not all open states are attainable, on average, from a single inactivated state.

#### 5.3.2. Small-Molecule BK Channel Activators

The calculation of entropy can also be useful in studies of selected channel modulators (as in this work). A good representative could be the activation of the BK channel by negatively charged, amphiphilic, small-molecule activators, as studied in [70]. These molecules can bind hydrophobically to the channel cavity and simultaneously drag water inside the cavity via their polar regions. The binding is possible as long as hydrophobic surfaces are present.

The dwell-time switching (observable at the patch-clamp time resolution of fractions of milliseconds) in this case will be dominated by the fluctuations in the selectivity filter and its dewetting process. (Other, short-lived fluctuations average out in the current recording). The different potential energy barriers for K^+^ ion entry to the pore, which depend upon the binding site of the activator, imply variability in the dwell-time scales for these processes, which will be reflected in the Shannon entropy.

Shannon entropy could be useful in this problem after the authors identified some bound states for the activator to check whether it can account for the measured entropy of the patch-clamp current. If not, further binding sites are to be searched for. Then, sample entropy (SampEn) could be used to estimate the number of pore conformations (including possible dewetted and recovered conformations) related to a particular binding site of an activator.

Further considering the methodology applied in this work, both PSD and EMD results provide insights into different aspects of the data. The relative PSD describes how the power of a signal is distributed across different frequencies in the presence of analyzed agents compared to the control data, and this is presented in the form of a continuous function over the frequency domain. The results of the PSD-based analysis were supplemented by entropy, which describes the variability in the power spectrum peaks related to Ca^2+^- and Que for subsequent bandwidths.

In contrast, EMD provides a discrete representation of the signal as a sum of IMFs, with each capturing time-localized oscillatory modes with potentially time-varying frequency and amplitude. Because, in this wor, we inspected the properties of a large IMFs population obtained for a wide set of signals (each set obtained at a fixed [Ca^2+^] and [Que]) across a range of frequencies, we focused on how the oscillatory patterns represented by IMFs grouped in frequency and amplitude (energy) domains at given conditions and neglected the details of the particular time-localization of oscillations within each of the analyzed signals (which could be useful in a detailed description of gating kinetics with tracked chronology). This strategy helped us in the separation of different frequency and relative power scales of single-channel current fluctuations for Que- and/or Ca^2+^-activated channels and non-stimulated controls, and to represent the results in a convenient manner, i.e., in the form of 2D histograms (Figure 7) or even easy-to-track clusters (Figure 1, Figure 8 and Figure 9).

In our paper, we present the results of the spectral and entropy-based analysis, as well as the EMD decomposition, to gain information about the ranges of signal frequency in which the most striking stimulus-specific changes in gating dynamics occur. A basic inspection of the experimental data, including the evaluation of open-state probability, provides fundamental, but approximate, information about the effects of a given stimulus on channel activity. The $p_{op}$ is a cumulative measure, which does not account for the nuances of conformational switching in the channel. Entropy, however, can differ depending on the type of conformational switching that accounts for a particular $p_{op}$ value. This is because entropy is sensitive to even delicate changes in the system’s complexity.

The (temporary) “switching fingerprint” depends on the biochemical microenvironment of particular channel conformation and allows for the indirect “measurement” of individual channel conformations. Furthermore, in this work, entropy was applied in PSD- and EMD-based approaches to describe the dispersion of the obtained results. Thus, the PSD and EMD analyses (within a shorter window length than the dwell time of the fastest macroconformation), complemented by appropriate entropy-based measures, can be used to recognize the stimulus-affected conformational states of the channel.

Our results can also be considered a useful constraint for refining Markovian ion channel models, where the obtained measures can be used for the optimal selection of model parameters. Furthermore, our approach may be useful for designing drugs that target a specific channel state. According to this idea, a drug should affect only a particular state of a particular type of on–off fluctuation that is identified as disease-related. In other words, a novel drug should inactivate only one conformation of a given entropic/spectral signature, rather than impair the overall channel operation, as the common active substances can.

This work represents the first application of EMD to patch-clamp data. EMD has already emerged as a powerful technique in the analysis of other electrophysiological signals, particularly in electrocardiography (ECG) and electroencephalography (EEG) applications [80,81,82,83]. As an adaptive and data-driven approach, EMD derives its decomposition solely from the intrinsic properties of the signal, eliminating the need for predefined basis functions. These characteristics make it especially well-suited for processing the nonlinear and nonstationary signals frequently encountered in biomedical research. We can see a noticeable gap in ion channel-oriented research regarding the application of advanced signal decomposition methods to patch-clamp recordings. We believe that patch-clamp data represent a unique niche where modern signal decomposition techniques, such as EMD, could uncover subtle dynamic features of channel gating and provide deeper insights into ion channel kinetics under different stimulation conditions.

## 6. Conclusions

The results of the entropic, spectral, and EMD analyses allow us to conclude that the Ca^2+^-related effects unarguably dominate in shaping the BK channel gating dynamics when considered alongside the effects of quercetin. Nonetheless, within the collected data, one can observe symptoms of their complex coupling: the administration of quercetin at a low concentration (∼10 μM) in the presence of Ca^2+^ results in a kind of competition between the agents. This observation allow quercetin to be considered as a powerful modulator of the calcium response of the BK channels. Conversely, the effects of Que activation at a high concentration (∼100 μM) are strongly alleviated in the presence of Ca^2+^ ions for unknown reasons. The applied methodology not only revealed alterations in gating complexity but also delineated the frequency ranges in which these effects were most evident, providing a basis for hypotheses on their mechanistic origin.

## Figures and Tables

**Table 1:** The open state probability ($p_{op}$) for experimental patch-clamp signals obtained at given Ca^2+^ and Que concentrations (denoted as [Ca^2+^], [Que]). The $p_{op}$ values are given as the mean ± standard error.

$p_{\mathrm{op}}$		[Que]		
		0	10 μM	100 μM
[Ca^2+^]	0	0.23 ± 0.05	0.23 ± 0.05	0.38 ± 0.08
	10 μM	0.83 ± 0.03	0.89 ± 0.02	0.90 ± 0.02
	100 μM	0.91 ± 0.01	0.85 ± 0.03	0.93 ± 0.01

**Figure A1:**
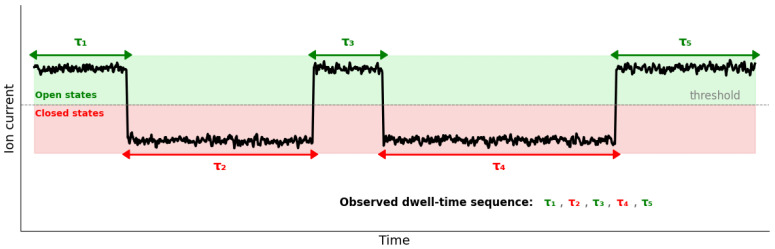
A schematic representation of the construction of the dwell-time series based on the signal channel recording. Threshold current separates open and closed states. Single-channel patch-clamp current amplitudes greater than the TC are classified as open states, while amplitudes equal to or below the TC are classified as closed states. The dwell-time series represents the durations ($\tau_{i}$s) of successive open and closed states.

## Data Availability

The research data are available on personal request.

## Abbreviations

The following abbreviations are used in this manuscript:

BK	Large-conductance voltage- and Ca^2+^-activated K^+^ channels
ECG	Electrocardiography
EEG	Electroencephalography
EMD	Empirical Mode Decomposition
FFT	Fast Fourier Transform
GR	cytoplasmic ‘Gating-Ring’ of the BK channel
HBE	Human Bronchial Epithelium
IMF	Intrinsic mode function
MD	Molecular Dynamics
mitoBK	Mitochondrial large-conductance voltage- and Ca^2+^-activated K^+^ channels
PG	pore-gate domain
$p_{op}$	Open state probability
PSD	Power Spectral Density
Que	Quercetin
SampEn	Sample entropy
VSD	Voltage Sensing Domain

## Appendix A. Shannon and Sample Entropy in the Analysis of Dwell-Time Series—An Extended Explanatory Description

In this work, we applied two types of information entropy (Shannon entropy and sample entropy) to characterize the data describing ion channel activity under different conditions. In order to make these measures readable and useful for a broad audience, we wish to provide some additional background related to their interpretation. Information entropy is typically expressed in bits, the basic units of information. Entropy tells us how many bits we need to encode all events within a time series (by assigning a unique symbol to each considered event, such as the dwell time duration). Naturally, the greater the diversity of events, the more bits are needed to distinguish them. Importantly, information entropy is a well-defined theoretical construct, and the occurrence of its well-defined units allows one to compare entropies across different phenomena (e.g., different ion channels) without scaling coefficients.

In this study, we first applied entropic analysis to the dwell-time series of the open (conducting) and closed (non-conducting) functional states of the channel. These series are typically obtained by first determining a threshold current (TC) that separates open and closed states. Single-channel patch-clamp current amplitudes greater than the TC are classified as open states, while amplitudes equal to or below the TC are classified as closed states. The dwell-time series was then constructed by measuring the durations of successive open and closed states. Thus, dwell time series (τ) represent how long the successive channel states last (as schematically presented in Figure A1).

Each value of the dwell-time series is represented by a floating-point number. Of course, the accuracy of this number is limited to the sampling rate ($10^{-4}$ s), which also introduces the problem of very short “missing events” (for which advanced theoretical statistical frameworks exist). Using dwell-time series instead of raw patch-clamp data or dichotomous binarized currents (i.e., a series of zeros and ones representing the length of the original patch-clamp recording, where, for each single-channel current, 1 was assigned to represent the open functional state and 0 represents a current value that was identified as closed functional state) offers clear advantages. Dwell-time series allow for a more compact representation of the data, effectively packing more information into shorter sequences. Moreover, the dwell-time series consists of the durations of the functional channel states that are modulated by conformational changes in the channel protein.

Now, let us simply rewrite the definition of Shannon entropy. If we consider a random variable *X* with possible outcomes $x_{i}$ and probabilities $p(x_{i})$, Shannon entropy is defined as follows:

(A1) $$H\left(X\right)=-\sum p\left(x_{i}\right)\log_{2}p\left(x_{i}\right)$$

Units are bits (when one uses base 2 of the logarithm), and this and can be interpreted as the average minimum number of bits needed to encode each symbol of the time series of random variables generated by this distribution. The more complex the distribution of a variable (the distribution of dwell times in our analysis), the higher the number of bits needed to encode each outcome (possible value of dwell-time length), and the larger the value of Shannon entropy. Thus, the Shannon entropy, when referring to the dwell times, describes the diversity of the dwell times, i.e., how many bits are required to encode the population of the measured dwell times. If all dwell times are equal, we do not need any information to encode this (this trivial situation would occur if all subsequent open- and closed-channel states last, e.g., 10 ms, so we would have the dwell-time series 10 ms, 10 ms, 10 ms, etc.). Then, the receiver simply a priori knows the sequence of dwell times. If there are two different, equiprobable dwell times, a single bit is enough (e.g., 10 ms, 5 ms, 10 ms, 5 ms, 10 ms, 5 ms; shuffling possible); four different, equiprobable dwell times (e.g., 1 ms, 5 ms, 10 ms, 7 ms, 10 ms, 5 ms, 7 ms, 1 ms); two bits, and so on. If the probability of a particular dwell time is low, we can encode it with some longer codes, leaving short codes to the frequently observed dwell time durations and obtaining an optimal average (typically fractional) number of bits for coding.

This background directly provides an interpretation of the Shannon entropy: it is low when the channel switching structure is limited (as in the highly activated channel state, where only long open dwell times occur, separated by short closed dwell times). Per this analogy, the same occurs in the resting state: only long closed dwell times occur, separated by short open dwell times. Thus, in the fully resting channel state (when open-state probability approaches zero) and fully activated channels (open state probability approaches 1), the entropy should have small values. When the channel is in the intermediate state, the entropy grows in a manner dependent upon the complexity of switching patterns (the more different the dwell time durations present in the record, the larger the increase in entropy)—these changes in entropy do not show a linear relationship with the open-state probability and depend on the gating machinery that is responsible for the structural richness of the dwell-time distribution. Thus, they provide new information about channel gating. One can characterize the structural complexity of a variable’s distribution by a single number—the Shannon entropy—which is straightforward to compute and can be directly compared across datasets obtained under different conditions.

To understand the differences and relations between Shannon entropy and another object of interest, the sample entropy, let us write the definition of sample entropy (SampEn) in a simplified way: For the chosen embedding dimension (e.g., *m* = 2) and the similarity threshold (e.g., *r* = 0.2σ, which is quite a typical tolerance level), one should calculate the following:

- The number of pairs of sequences of length m that match within tolerance *r*

- The number of pairs of sequences of length m+1 that match within tolerance *r*, and finally compute

SampEn = −log((probability that two sequences match for m+1 points)/(probability that two sequences match for *m* points)

(A2) $$\mathrm{SampEn}=-log(\frac{\mathrm{probability}\;\mathrm{that}\;\mathrm{two}\;\mathrm{sequences}\;\mathrm{match}\;\mathrm{for}\;m+1\;\mathrm{points}}{\mathrm{probability}\;\mathrm{that}\;\mathrm{two}\;\mathrm{sequences}\;\mathrm{match}\;\mathrm{for}\;m\;\mathrm{points}})$$

The probability under the logarithm in this expression could be intuitively written as follows:

(A3) $$\frac{p(m+1)}{p(m)}=\frac{\left[p(m)p(m+1|m)\right]}{p(m)}=p\left(m+1|m\right),$$

i.e., it is a conditional probability of continuation (symbol with an index m+1) if the sequence started with *m*.

Sample entropy is a metric that quantifies the complexity and regularity of time series data. It measures the likelihood that similar patterns of data points remain similar when one more point is added, within a given tolerance. Basically, sample entropy is a similar concept to Shannon entropy, but the events analyzed in this approach relate to the continuation of a sequence of dwell times, not to a dwell time per se—in SampEn, we limit ourselves to dwell times that occur after a particular dwell time duration. For example, after a long opening, a channel may tend to turn into a short closing rather than into the state of any dwell time duration found in the series. The sample entropy will be low in such cases, even though the Shannon entropy of all dwell times may be large. In the definition of SampEn, such entropies of “continuation” are averaged over all initial dwell times, which may somewhat complicate the understanding of the picture. Nevertheless, please remember that within these dwell-times, SampEn is based on the conditional duration of a dwell time provided that the previous dwell time had a specific duration.

If the dwell-time series is regular, for example, patterns tend to repeat (e.g., **10 ms, 2 ms**, 4 ms, 6 ms, **10 ms, 2 ms**, 2 ms, 3 ms, **10 ms, 2 ms**, 4 ms, 6 ms, **10 ms, 2 ms**), the continuation probability is high, and SampEn should be relatively low. If the dwell-time series is irregular (e.g., 1 ms, 5 ms, 10 ms, 7 ms, 10 ms, 4 ms, 2 ms, 102 ms, 1 ms, …, 1 ms, 33 ms, …, 5 ms, 1 ms, …, 10 ms, 62 ms, …, 7 ms, 2 ms, …, 4 ms, 21 ms, …, 2 ms, 1 ms, …, 102 ms, 46 ms, …), the continuation probability drops (there are alternatives) SampEn should be relatively high. Shannon entropy describes the complexity of the distribution of a given variable as a whole. In turn, SampEn describes the complexity of the sequences of a given variable and the tendency for patterns to occur within the series of that variable. Thus, they refer to different features of the signal.

It is worth mentioning in the context of ion channel-oriented research that Shannon or sample entropy are not a better or worse characteristic than, for example, the open state probability or the dwell time distribution plot. They provide complementary information. Importantly—at least in the case of Shannon entropy—this information is normalized to units of bits, while it is very common that various measures display proportionality to some effects with an unknown (and changing from measurement to measurement) proportionality coefficient. Shannon entropy is exhibited as a single number that describes the overall effect of a given stimulus on the dwell-time distribution complexity. The inspection of the Shannon entropy and SampEn changes with a given channel stimulation allows for the formulation of hypotheses that may extend the picture of the functional consequences of channel–ligand(s) interactions for the gating phenomenon, as broadly discussed in the dedicated Section 5.2 and Section 5.3 of this article.
